# The PtDPL1*/PtGAMYB-PtLEAFY* module regulates pollen fertility and flowering time in *Pinus tabuliformis*

**DOI:** 10.3389/fpls.2025.1711944

**Published:** 2026-03-16

**Authors:** Huili Wang, Jingxing Zhang, Chengcheng Zhou, Zhiyuan Jiao, Yousry A. El-Kassaby, Wei Li

**Affiliations:** 1 State Key Laboratory of Tree Genetics and Breeding, National Engineering Research Center of Tree Breeding and Ecological Restoration, College of Biological Sciences and Technology, Beijing Forestry University, Beijing, China; 2 Department of Forest and Conservation Sciences, Faculty of Forestry, University of British Columbia, Vancouver, BC, Canada

**Keywords:** cone development, *Pinus tabuliformis*, PtDPL1, *PtGAMYB*, *PtLEAFY*

## Abstract

Reproductive development is a crucial stage in the plant life cycle. The *GAMYB* transcription factor plays an important role in regulating stamen development. Here, *PtGAMYB* was identified as part of the GA signal transduction pathway of *Pinus tabuliformis* and demonstrated to play a role in male reproductive development. Subcellular localization assays revealed that the *PtGAMYB* protein is localized in the nucleus. Both RNA-seq and fluorescence *in situ* hybridization demonstrated that *PtGAMYB* is specifically and highly expressed in the male cones and pollen of *Pinus tabuliformis*. Heterologous expression of *PtGAMYB* in *Arabidopsis thaliana* delayed flowering time, reduced pollen germination rates, and impaired the seed set. However, these phenotypes could be restored by exogenous GA_4 + 7_ treatment. Additionally, binding and activation analyses indicated that *PtGAMYB* acts as an upstream regulator of *PtLEAFY*. Further studies determined that *PtGAMYB* interacts with PtDPL1 protein to enhance the inhibition of *PtLEAFY*, ultimately regulating male cone formation in *Pinus tabuliformis*. Our findings establish a novel PtDPL1/*PtGAMYB-PtLEAFY* regulatory network and provide new insights into the reproductive mechanisms in conifers.

## 1 Introduction

Inflorescence and flower development are critical processes for ensuring species survival and evolutionary success in vascular plants, serving as foundational models for investigating the interplay between reproductive organ evolution and phylogenetic relationships (Hanzawa et al., 2005, Teotia and Tang, 2015). Because angiosperms and gymnosperms exhibit fundamental divergences in reproductive strategies (Sederoff, 2013; Qu et al., 2025), the primary reproductive organs in conifer are macrosporophyll and microsporophyll (Taylor and Taylor, 2009; Wang et al., 2010; Zhou et al., 2025). Despite extensive characterization of floral development mechanisms in angiosperms, current models fail to account for the unique reproductive and wood-production requirements of conifers. The regulatory mechanisms governing reproductive development in gymnosperms therefore remain largely unknown.

Gibberellin (GA) is an important phytohormone that regulates floral development (Qi et al., 2014; Hu et al., 2018). The *MYB* gene family, one of the largest gene families in eukaryotes (Lipsick, 1996), plays an important role in reproductive development (Dai et al., 2007; Nakano et al., 2015; Lv et al., 2017; Lloyd et al., 2017). Within this family, a special class of *GAMYB* subfamily genes (*R2R3-MYB* family) specifically responds to GA regulation and is involved in male development and seed formation (Kaneko et al., 2004; Millar and Gubler, 2005). In *Arabidopsis*, the *GAMYB* gene expression is post-transcriptionally regulated by a set of microRNAs (miRNAs), namely, miR159 (*miR159a, miR159b, miR159c*) (Allen et al., 2007; Aya et al., 2009). Previous studies have demonstrated that *GAMYB* is involved in regulating tapetum development in the anther and mediating GA-induced flowering response (Miyuki,2004; Kaneko et al., 2004; Murray et al., 2006; Liu et al., 2010). GA promotes the expression of *GAMYB* and *SOC1* by degrading DELLA proteins, which in turn activates transcription of the downstream gene *LEAFY* to induce flowering (Millar and Gubler, 2005; Li et al., 2016a; Wang et al., 2018). Intriguingly, while *GAMYB* homologs are present in both *Bryophyta* and *Selaginella*, functional GA-mediated regulatory pathways are exclusively observed in *Selaginella*. This evolutionary divergence suggests that although GA metabolic pathways emerged early in land plant evolution, their functional specialization in reproductive development became prominent in higher plants (Aya et al., 2009; Aya et al., 2011).

In the GA-DELLA-*GAMYB* flowering regulatory pathway, GA-mediated degradation of DELLA proteins derepresses *GAMYB* transcription, thereby initiating floral transition (Sun, 2011; Kwon et al., 2015; Li et al., 2016b). In barley, *HvGAMYB* is predominately expressed in anthers, where its overexpression causes dose-dependent phenotypic abnormalities, including shortened anthers, impaired dehiscence, and complete male sterility (Murray et al., 2006). In tomato, *SlMYB33* influenced flowering and pollen maturation through regulating the expression of genes involved in flowering and sugar metabolism (Zhang et al., 2020). Similarly, in rice, *OsGAMYB* displayed distinct spatial expression in shoot apical meristems, stamen primordia, and tapetal layers; its knockout results in defective pollen wall formation and aborted microspore development (Aya et al., 2009). The interaction between *GmGBP1* and *GmGAMYB* directly activated *GmSAUR*, thereby promoting early maturity in soybean and increasing plant height (Sun et al., 2023). *LoMYB33* plays a crucial role in pollen development and formation in Lily (Liu et al., 2021). Collectively, these findings highlight the essential role of the *MY*B gene family in both floral transition and flower development. However, although several transcription factors associated with the stamen development have been characterized in angiosperms, the mechanisms of reproductive regulation in conifers, particularly those involving *GAMYB* in *Pinus tabuliformis*, remain largely unknown.

Comparative genomic studies have identified two evolutionarily conserved *GAMYB* paralogs in gymnosperms, exhibiting low sequence divergence and high structural conservation (Jiang et al., 2019; Nemesio-Gorriz et al., 2017; Li et al., 2013a). The possible targets of miR159a were *TcMYB35*, all of which were clustered in the S18 subgroup, which is consistent with previous studies in *Arabidopsis* (Hu et al., 2020). In *Ginkgo biloba*, *GbMYB35* had the higher expression in stamens, indicating that it may be involved in the differentiation of staminate flowers (Liu et al., 2017). The post-transcriptional regulation of *LaMYB33* by miR159 participated in the maintenance of embryogenic or non-embryogenic potential and somatic embryo maturation in *Larix kaempferi* (Li et al., 2013b). Furthermore, the functional conservation of GA signaling components is supported by the confirmed GA-GID1-DELLA regulatory axis in *Pinus tabuliformis* (Du et al., 2017). Despite these advancements, critical knowledge gaps persist regarding *GAMYB* function in gymnosperms. To date, no systematic investigation has elucidated their roles in conifer reproductive timing or pollen development, including the putative *PtGAMYB* in *Pinus tabuliformis.*

Here, we isolated a nuclear-localized transcription factor, *PtGAMYB*, from *Pinus tabuliformis*. Its expression was detected mainly in male cone and pollen. Heterologous overexpression of *PtGAMYB* in *Arabidopsis thaliana* induced dose-dependent reproductive defects, including delayed flowering and reduced pollen viability, phenotypes that were partially rescued by exogenous GA_4 + 7_ application. In addition, *PtGAMYB* was found to interact with PtDPL1 and co-regulate downstream *PtLEAFY* expression, in turn affecting male cone development. Collectively, these findings provide new insights into the *PtGAMYB* regulatory network-mediated male cone development in *Pinus tabuliformis*.

## 2 Materials and methods

### 2.1 Plant material and sample collection

Research samples were collected from P. tabuliformis Seed Base of Qigou State Forest Farm in Pingquan City, Hebei Province (40°99′N, 118°45′E, elevation 560 m), with six biological replicates selected for each tissue sample. Sampling was uniformly conducted at noon. Samples included needles, roots, vegetative buds, and male and female cones from mature trees. Additionally, male and female cones at different developmental stages were collected from Beijing Forestry University (40°0.0135′ N, 116°34.535′ E, 43.5 m elevation). Sampling spanned from September 2012, when male and female cones became distinguishable, to April 2013, when male and female cones reached maturity (**Supplementary Figure S1**). Tissue samples covered six developmental stages. Transcriptome data for these samples (**Supporting Information Tables S3** and **S4**) were provided by Niu Shihui, with detailed sampling protocols described in Niu et al. (2016).

In this study, the Arabidopsis wild-type plants are in the *Columbia* (*Col-0*) genetic background. AlArabidopsis and Nicotiana benthamiana plants were grown in the soil under an artificial growth chamber at 22°C and 50% relative humidity, with a 16-h light/8-h dark photoperiod.

### 2.2 Sequence analysis, alignment, phylogenetic analysis, and expression pattern analysis of *PtGAMYB*

Using the *AtMYB33* sequence as a reference to NCBI (https://www.ncbi.nlm.nih.gov/), we retrieved the *PtGAMYB* (E-value <1^e−10^) by using BLAST(blast-2.6.0+) software. Basic information for the *PtGAMYB* gene is queried via ExPASy (http://www.expasy.org/tools/). Multiple sequence alignment of *PtGAMYB* with multispecies GAMYB was performed using ClustalX (http://www.clustal.org/clustal2/) and GENEDOC (GENEDOC 2.7.0). The 15 protein sequences were predicted using the maximum likelihood model (Maximum Likelihood model), and a phylogenetic tree was constructed using MEGA.X software; MYBs motifs were predicted by MEME (http://meme-suite.org) and CD-Search (https://www.ncbi.nlm.nih.gov/cdd/) and visualized using TBtools.

Based on the *Pinus tabuliformis* transcriptome data, gene expression levels were calculated by RSEM (http://deweylab.github.io/RSEM/) (Qu et al., 2025), and the expression levels of individual genes were converted to TPM (transcripts per million) values. Expression pattern heatmaps were drawn by TBtools (Chen et al., 2020), and the TPM values were log2 processed. Differential expression analysis was performed using DESeq2 to screen for differentially expressed genes between samples based on the criteria of log2 |fold change| ≥ 1 and adjusted p-value <0.05.

### 2.3 *PtGAMYB* fluorescence by *in situ* hybridization

The collected samples were quickly stored in DEPC fixative for 12 h and then embedded in paraffin by alcohol dehydration; the cut samples were placed at 62°C for 2 h, followed by xylene I, xylene ethanol I, 85% alcohol, and 75% alcohol, for 15 min, and finally washed with DEPC. The washed samples were boiled in repair solution for 10–15 min, cooled at room temperature, digested with protease K (20 μg/mL) for 25 min, washed three times using PBS for 5 min each, and incubated at 37°C for 1 h; the solution was removed; and 6 ng/μL of probe hybridization was added at 37°C overnight. Two saline-sodium citrate (SSC) buffer was washed for 10 min, 1 SSC buffer in the same conditions, washed twice for 5 min, and finally washed with 0.5 SSC for 10 min at room temperature, followed by DAPI staining and microscopy photography. Primers are shown in Supporting Information **Supplementary Table S1**.

### 2.4 Subcellular localization analysis

The *PtGAMYB* gene was cloned into the *PBI121-EGFP* vector into *Agrobacterium* LBA4404. *Agrobacterium* cells were cultured, collected, and resuspended in suspension (150μM acetosyringone, 10 mM MgCl_2_, and 10 mM MES) into tobacco leaves using a syringe. Three days after infiltration, images were taken using a Leica (TCS-SP8) confocal microscope. Primers are shown in Supporting Information **Supplementary Table S1**.

### 2.5 Yeast two-hybrid assay

The obtained *PtGAMYB* with a PtDELLA full-length sequence was inserted into pGBKT7 and pGADT7 vectors. Y2H assay was transformed using the Yeastmaker Yeast Transformation System 2 (Clontech), as previously described (Clontech Code No. 630439). After self-activation assays, transformation cultures were performed on SD/-Leu-Trp solid medium for 3 days and used for interaction detection using SD/-Leu-Trp-His-Ade medium. pGADT7-T and pGBKT7-lam were co-transformed into positive and negative controls, respectively. Primers are shown in Supporting Information **Supplementary Table S1**.

### 2.6 BiFC assay

BiFC expression vectors pSPYNE-*PtGAMYB*, pSPYCE-PtDPL1, and pSPYCE-PtDPL3 were constructed by seamless cloning and transformed into DH5α (weidibio, Beijing, China). After selecting the single clones and extracting the plasmids, preliminary enzyme digestion was verified, and the correctly verified plasmids were sent to Beijing Huada Bioengineering Company for sequencing. The successfully sequenced plasmid was transformed into *Agrobacterium* GV3101 (Weidi Biotech) and positively identified by PCR. After centrifugation to collect the bacteriophage, osmotic solution was added to adjust the *Agrobacterium* cell OD_600_ value to 0.8-1.0. Then, the bacterial solution was mixed according to the equal volume ratio of the experimental combinations (1:1 v/v) and gently pressed into the dorsal side of the tobacco leaf with a needleless syringe to avoid the leaf vein as much as possible. After 48 h, the yellow fluorescent protein and its distribution position in the epidermal cells of the tobacco were observed by laser confocal microscopy. Primers are shown in Supporting Information **Supplementary Table S1**.

### 2.7 Co-immunoprecipitation

The pEarleyGate201-*PtGAMYB*-YN and pEarleyGate202-PtDPL1-YC expression vectors were constructed, and the plasmids of the successfully detected positive strains were transferred into *Agrobacterium* GV3101 and positively cloned by *Agrobacterium* solution using PCR technology. After overnight propagation of the successful *Agrobacterium* solution tested, according to group 1: pEarleyGate201-*PtGAMYB*-YN pEarleyGate202-PtDPL1-YC; group 2: pEarleyGate201-*PtGAMYB*-YN; and group 3: order of the pEarleyGate202-PtDPL1-YC, the bacterial mixture was mixed to prepare the infection solution, injected into the back side of the tobacco leaves. A fraction of the protein extracted supernatant as the input group, and an equal volume of 2 SDS loading dye was also added, with the SDS-PAGE serving as a control. The remaining protein extraction supernatant was added to the pre-equilibrated anti-HA-tag magnetic beads of approximately 10 μL, as the IP group. The results of protein identification were analyzed between In-put and IP groups. Primers are shown in Supporting Information **Supplementary Table S1**.

### 2.8 Construction of transgenic plants

The *PtGAMYB* cloning vector and the pBI121-*PtGAMYB-*EGFP overexpression carrier were constructed using the seamless cloning method (Biomed, 2×Seamless Cloning Mix CL117-02). The *E. coli* competent DH5α was transformed, the positive strains with successful sequencing were expanded, plasmids were extracted and transferred to *Agrobacterium* GV3101, and positive clonal identification of *Agrobacterium* bacterial solution was carried out by PCR. The 35S:: *PtGAMYB* overexpressing transgenic *Arabidopsis* lines were obtained by flower immersion transformation using *Arabidopsis Col-0*, screened on MS plates containing kana (50 mg/mL). The phenotypes of the transgenic plants were verified in at least three independent transgenic lines and identified by sequencing from each generation of positive seedlings. All *Arabidopsis* plants were grown in artificial climate boxes with light conditions of LD (16 h light/8 h dark, 23°C). Primers are shown in Supporting Information **Supplementary Table S1**.

### 2.9 Yeast one-hybrid assay

The complete coding sequence (CDS) of *PtGAMYB* was amplified utilizing reverse transcription-polymerase chain reaction (RT-PCR) and subsequently ligated into the pB42AD vector to produce the construct pB42AD-*PtGAMYB*. Genomic DNA served as a template for PCR amplification of the promoter regions located upstream of the transcription start sites of PtLFY (region from −638 to −275 bp). These promoter fragments were then cloned into the pLacz2µ vector (Clontech) to generate the recombinant reporter plasmids pLacZ2µ-PtLFY. To verify the interactions, the two fusion plasmids were co-transformed into the yeast strain EGY48 following the protocols detailed in the Yeast Protocols Handbook (Clontech). Four stringent negative controls were established: pB42AD + pLacz2µ, pB42AD + pLacZ2µ-PtLFY, pB42AD-*PtGAMYB* + pLacz2µ, and pB42AD-PtDAL1 + pLacz2µ-pLacZ2µ-PtLFY. The yeast transformants were cultured on SD/-Trp/-Ura medium. After incubation at 28 °C–30°C for 3 to 5 days, six clones were selected and grown in liquid SD/-Trp/-Ura medium while shaking at 200 rpm and 28°C for 24–36 h. Positive clones were identified, and 2 μL of the culture was spotted onto synthetic dextrose plates lacking uracil and tryptophan but supplemented with 20 μg/mL X-gal (Wu et al., 2017). The plates were incubated in the dark for 3 to 5 days to monitor the color development of the yeast colonies. Primers are shown in Supporting Information **Supplementary Table S1**.

### 2.10 Luminescence assay

The CDS of *PtGAMYB* was cloned into the pGreen 62-SK vector serving as the effector plasmid. For the promoter regions, sequences spanning from −638 to −275 bp for PtLFY were amplified and cloned into the pGreen II 0800-LUC vector acting as the reporter plasmid. The resulting recombinant vectors along with negative control vectors were introduced into *A. tumefaciens* strain GV3101 carrying the helper plasmid pSoup. The transformed *A. tumefaciens* cultures were grown in Luria-Bertani broth supplemented with appropriate antibiotics. After growth, the bacterial cultures were harvested by centrifugation at 4,500 rpm for 10 min and resuspended in infiltration buffer (10 mm MES, 10 mm MgCl2, 0.2 mm acetosyringone, pH 5.6) to achieve an optical density at 600 nm (OD_600_) of approximately 1.0 (Zhang et al., 2021). For *N. benthamiana*, leaves of plants aged around 4 weeks (having six to eight true leaves and not yet flowering) were infiltrated with a mixture of the effector and reporter vectors. Post-infiltration, the plants were initially kept in darkness for 12 h followed by growth under a 16-h light/8-h dark photoperiod for an additional 36 to 48 h. Prior to imaging, d-Luciferin (10 μM) was applied to the leaves, and photographs were taken using a Tianneng 5200 multimolecular imaging system (Song et al., 2025; Zhou et al., 2025). Primers are shown in Supporting Information **Supplementary Table S1**.

### 2.11 GA treatment test and pollen germination test

In order to explore the role of *PtGAMYB* in the GA signaling pathway, 0.2 µmol of GA_4 + 7_ and clear water (CK) were applied to the transgenic plants of *PtGAMYB*, and the leaves and flower heads of *Arabidopsis thaliana* were sprayed at the early stage of flower bud development. The GA_4 + 7_ concentration and specific procedures were referenced from Liu et al. (2022) and Liu et al. (2025). Every week in the morning at 11-12:00, the plants were sprayed twice. The pollen of *PtGAMYB* transgenic plants was further treated with GA_4 + 7_, and a glass rod was dipped in a small medium (100g/L sucrose, 10mg/L boric acid, 5g/L agar), spread on the slide, and placed in a Petri dish lined with moist filter paper. The pollen collected at about 10:00 in the morning was evenly spread on the medium, and the Petri dish was sealed to maintain humidity and placed in a 24 °C light and constant temperature incubator for cultivation and observed every 4 h. After 8 h, the pollen germination rate of wild-type *Arabidopsis* can reach 50%-70%.

### 2.12 Statistical analyses

The data were organized using Excel 2019 software (Microsoft, Redmond, WA, USA), SPSS23.0 (IBM, New York, NY, USA) as used for statistical analysis at p < 0.05 with Tukey’s test. The graphs were plotted using GraphPad Prism Software (8.0), and the data were presented as the mean ± standard deviation, Values with different letters above the bars are significantly different at p < 0.05.

## 3 Results

### 3.1 Characterization of PtGAMYB in *Pinus tabuliformis*

**Figure 1:**
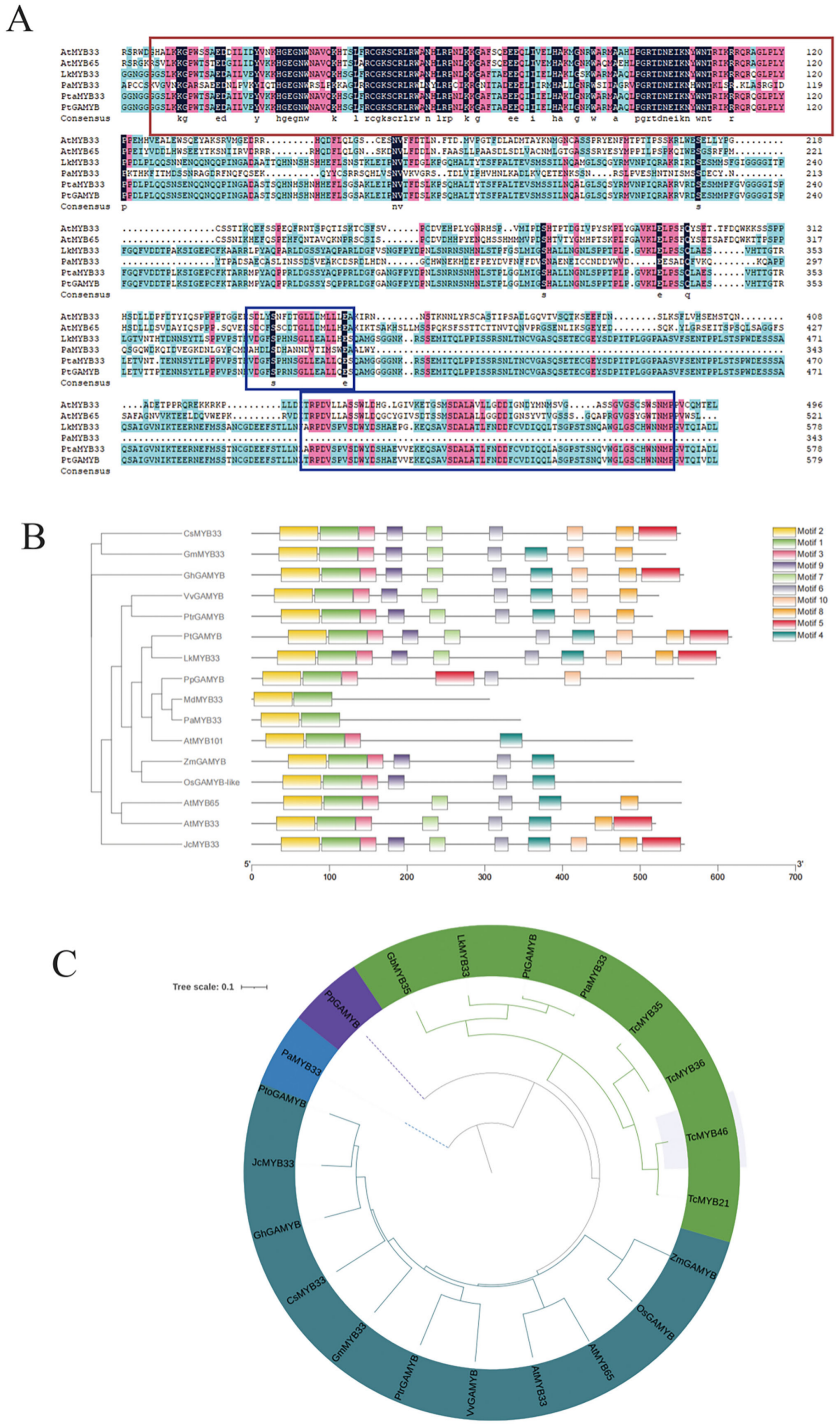
Physicochemical characteristic and sequence feature of *PtGAMYB* **(A)** Multiple-sequence alignment and motif compositions of *GAMYB* proteins in different species. The red region in A is the R2R3 domain, and the blue region is the Box1, Box2, and Box3 conserved regions. **(B)** Motif analysis of *GAMYB* genes in diverse species. **(C)** Phylogenetic analysis of *GAMYB* genes in diverse species.

The *PtGAMYB* gene (GenBank: Pt5G01300.1), mapped to chromosome 5 (45,562,533-45,565,332 bp) of *Pinus tabuliformis*, exhibited a canonical three-exon/two-intron structure (Supporting Information **Supplementary Figure S1**). Full-length cDNA cloning from male cone tissues revealed a 1,857-bp open reading frame encoding a 618-amino-acid protein with a predicted molecular weight of 66.65 kDa and an isoelectric point of 5.50 (Supporting Information **Supplementary Table S2**). The multiple-sequence alignment from multiple species showed that *PtGAMYB* contained a conserved R2R3 domain at the N-terminus and three *GAMYB*-specific conserved structures Box1, Box2, and Box3 (**Figure 1A**), indicating that *PtGAMYB* belonged to the *R2R3-MYB* family. Notably, these non-canonical variations in conserved boxes paralleled those observed in angiosperm homologs (*GmMYB33, AtMYB65*, and *ZmGAMYB*), suggesting evolutionary divergence in motif architecture while maintaining functional conservation (**Figure 1B**).

Phylogenetic clustering positioned *PtGAMYB* within the conserved *GAMYB* subfamily, exhibiting complete sequence identity (100%) with *Larix kaempferi PkMYB33*, while showing 90% homology to *Arabidopsis* reproductive regulators *AtMYB33/65* versus 74% to ovule-specific *AtMYB101* (**Figure 1C**). In addition, promoter cis-element analysis (2-kb upstream region via PlantCARE) identified eight regulatory modules: CGTCA (MeJA-responsive), TCCC (light-responsive), LTR (low-temperature), MBS (drought-inducible), RY-element (seed-specific), HD-Zip 1 (meristem differentiation), O2-site (metabolic control), and anaerobic induction elements (Supporting Information **Supplementary Figure S3**). These results showed that *PtGAMYB* may integrate multiple signaling pathways (hormonal, environmental, and developmental) to coordinate reproductive processes in conifers.

### 3.2 *PtGAMYB* is a key regulator of male cone development in response to GA

**Figure 2:**
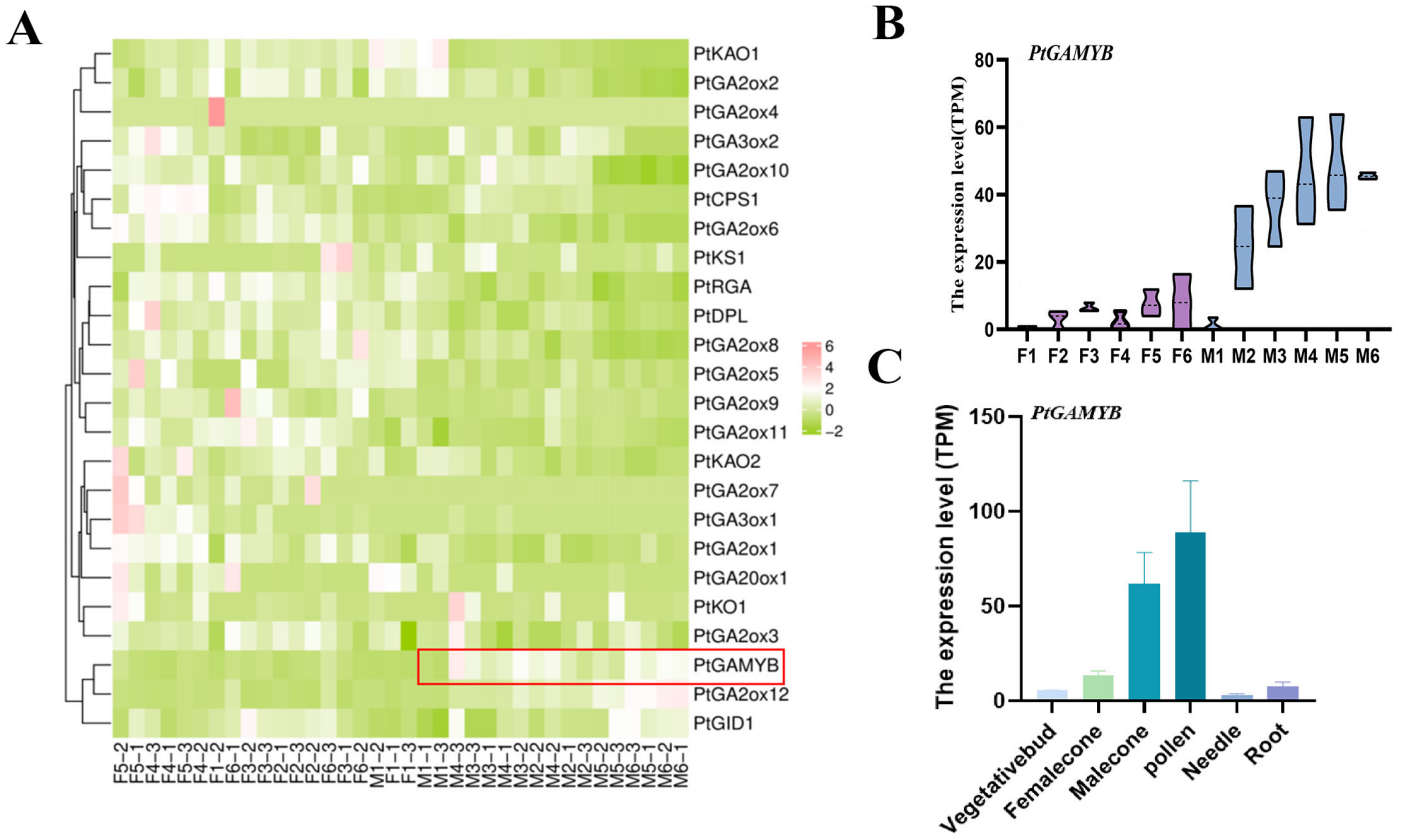
Transcriptome profiles of *PtGAMYB* in *Pinus tabuliformis.* **(A)** Differential gene expression of GA synthesis and signal transduction pathway in *Pinus tabuliformis*. Red box: expression of *PtGAMYB* during the developmental stage of the male cone. **(B)** Transcriptome profiles of *PtGAMYB* at different stages of reproductive development. **(C)** Transcriptome profiles of *PtGAMYB* under vegetative bud, female cone, male cone, pollen, needle, and root.

Through comparative genomics analysis using *Arabidopsis* GA biosynthesis and signaling pathway components as reference, 46 orthologous genes associated with GA metabolism in *Pinus tabuliformis* cone developmental transcriptomes were identified. Among these, 26 genes exhibited significant differential expression across developmental stages. Notably, differential expression screening revealed partial conservation of GA-responsive regulators, with the majority maintaining basal expression levels throughout cone development. Further analysis revealed that multiple genes regulating GA flower development were upregulated in the female cone, whereas only *PtGAMYB* was specifically upregulated and expressed in male cone (**Figure 2A**).

The expression levels of *PtGAMYB* in *Pinus tabuliformis* cone at different development stages were analyzed (**Figure 2B**). Transcript levels progressively increased during male cone maturation and increased gradually with developmental stages. The analysis of RNA-seq read counts indicated that the expression of *PtGAMYB* was higher in male cone and pollen (**Figure 2C**), indicating that *PtGAMYB* expression exhibited both developmental stage dependency and male cone specificity, suggesting its potential regulatory role in late-stage microgametogenesis.

### 3.3 *PtGAMYB* is localized in the nucleus and specifically expressed in male cone

**Figure 3:**
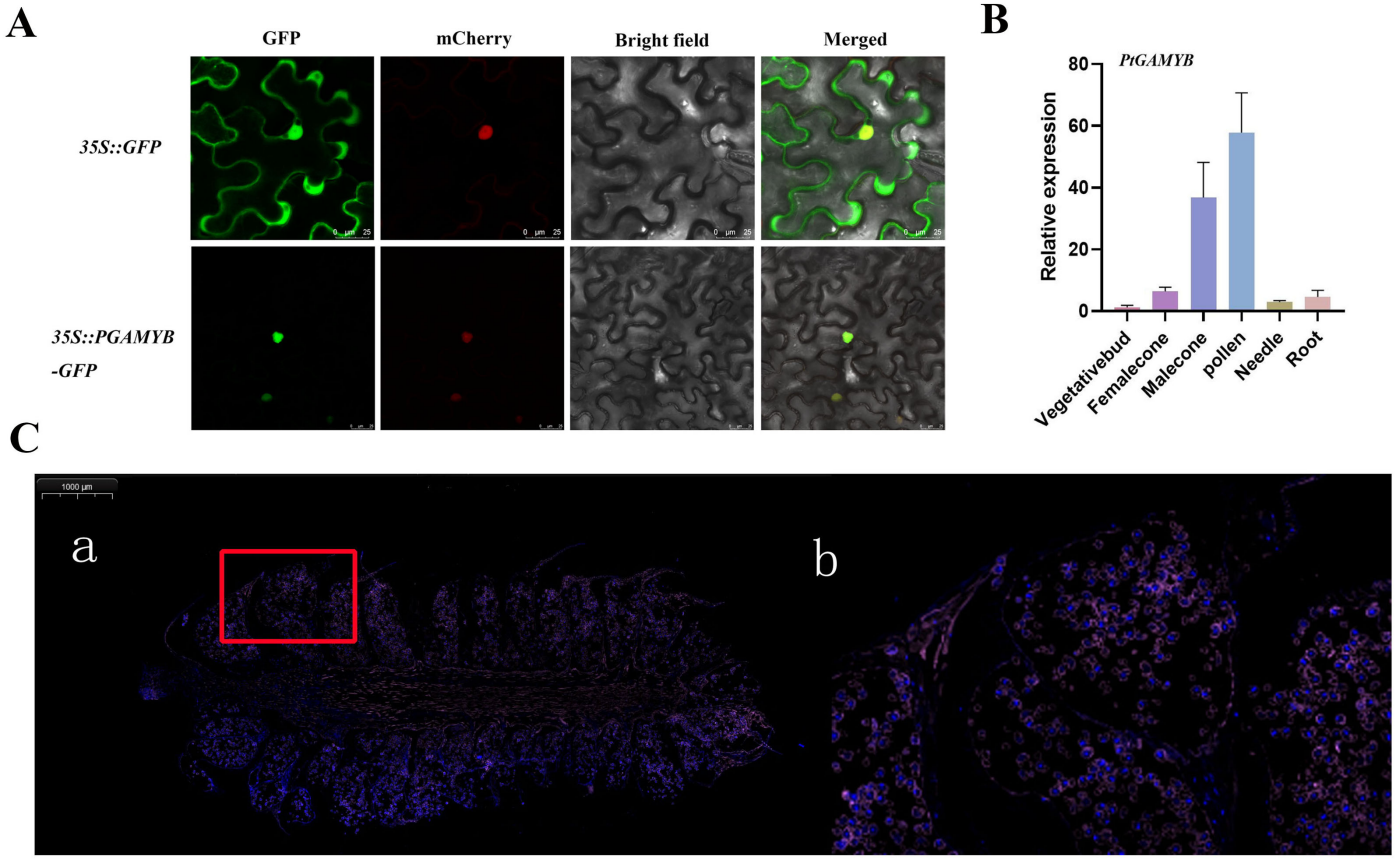
The expression patterns of *PtGAMYB* in the male cone. **(A)** Subcellular localization of *PtGAMYB*. The green fluorescence signal is the location result of p35S::-GFP and p35S:: *PtGAMYB*-GFP. The red fluorescent signal mCherry is nuclear-localized, Bright is the Bright field, and Merge is the superposition fluorescence signal (Yellow). Bars = 25 µm. **(B)** Relative expression of *PtGAMYB* in various tissues by RT–qPCR. The samples from left to right are as follows: vegetative bud, female cone, male cone, pollen, needle, and root. Data are means ± SE from three replication. **(C)** Fluorescent *in situ* hybridization of the *PtGAMYB* gene in *Pinus tabuliformis.*, b shows the red area in Figure (a) Bars = 100 µm.

To determine the localization pattern of *PtGAMYB* in cells, the CDS region of *PtGAMYB* was inserted into the pBI121-eGFP vector and then transiently expressed by infiltrating tobacco. In the cell structure, the nuclear signal p35s:: GFP expression vector chloroplast signal GFP was used as a positive control, and *PtGAMYB* was fluoresced green in the nucleus (**Figure 3A**). RT-qPCR revealed significant differences in *PtGAMYB* expression between tissues. *PtGAMYB* was highly expressed in male cone and pollen, whereas it was expressed at very low levels in vegetative bud, female cone, needles, and roots (**Figure 3B**); results are consistent with RNA-seq. Subsequently, fish fluorescence *in situ* hybridization assay was performed on the M5 stage at the later stage of the male cone; the nuclei (DAPI staining) showed blue color under UV. It was found that *PtGAMYB* was specifically expressed in the microspores and pollens (**Figure 3C**); its expression is much higher in pollens than in other locations, indicating that the function of *PtGAMYB* in the male cone is associated with pollen formation. These data strongly support the possible involvement of *PtGAMYB* in male cone development of *Pinus tabuliformis*.

### 3.4 Overexpression of *PtGAMYB* leads to delayed flowering in *Arabidopsis*

**Figure 4:**
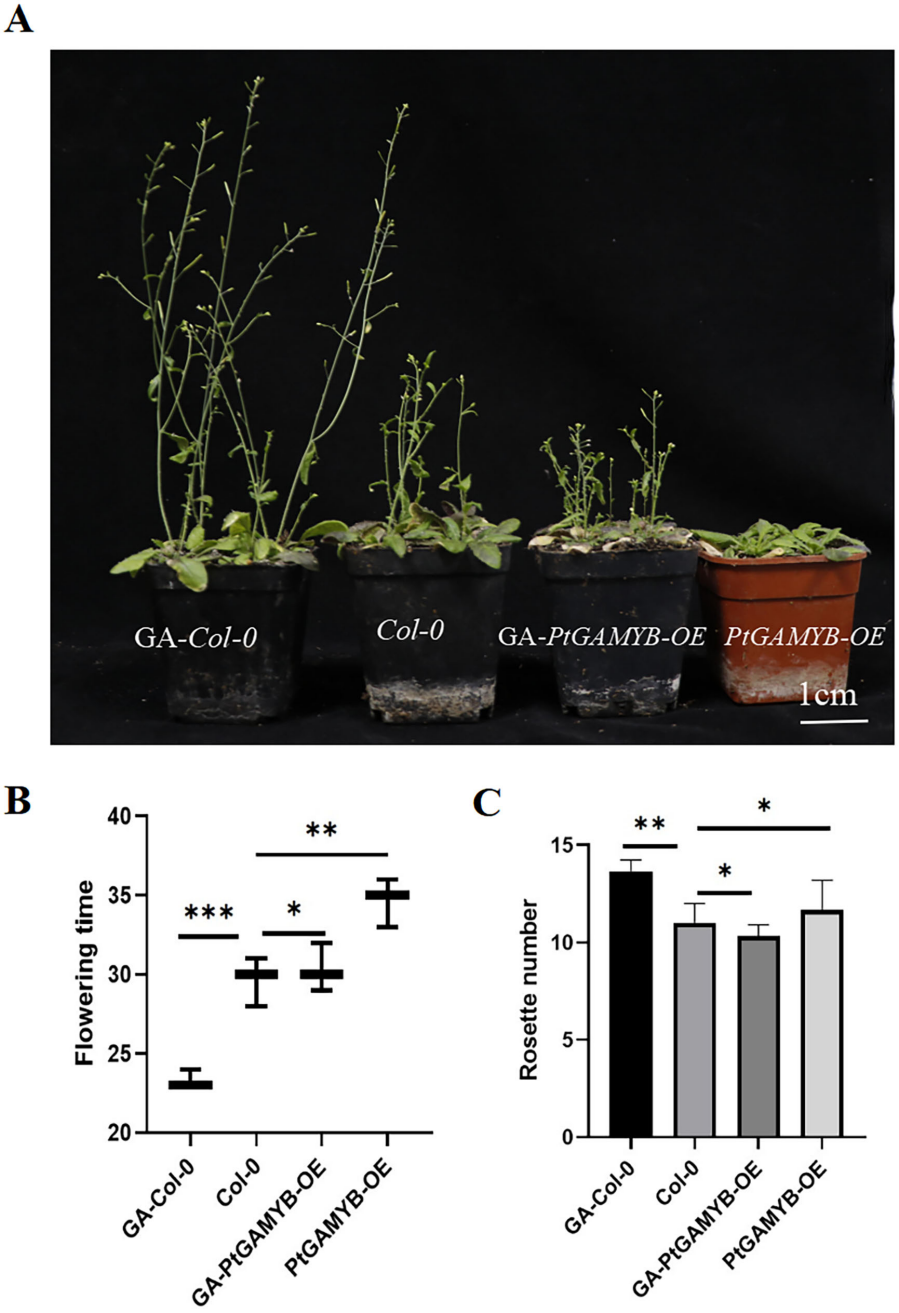
Transgenic phenotypic observations of *PtGAMYB* in *Arabidopsis*. **(A)** Transgenic phenotype of *PtGAMYB* in *Arabidopsis*. **(B)** Statistics of flowering time in transgenic lines of GA-*Col-0, Col-0*, *PtGAMYB-OE*, and GA-*PtGAMYB-OE*. **(C)** Statistics of rosette leaves in transgenic lines of GA-*Col-0, Col-0*, *PtGAMYB-OE*, and GA-*PtGAMYB-OE*. The values represent the means ± standard deviations (n = 3 plants for each replicate). ^∗^P < 0.05; ^∗∗^P < 0.01, ^∗∗∗^P < 0.001 (Student’s t-test).

In order to validate the regulatory function of *PtGAMYB* in reproductive development, *PtGAMYB*-overexpressing transgenic *Arabidopsis* lines were generated and planted (Supporting Information **Supplementary Figure S4**). In *Arabidopsis*, overexpression of *PtGAMYB* transgenic plants exhibited delayed flowering phenotype under LD conditions (**Figure 4A**). *PtGAMYB-OE* lines flowered on average 4.1 days later than *Col-0* (**Figure 4B**). In addition, the number and size of rosette leaves in *PtGAMYB-OE* lines were not significantly different from that of *Col-0* (**Figure 4C**).

We performed a GA-treated phenotypic recovery assay on the *PtGAMYB-OE* line. Notably, the late-flowering phenotype of *PtGAMYB-OE* lines was completely or partially restored by exogenous GA_4 + 7_ treatment (**Figure 4A**). Under equivalent growth conditions, the *PtGAMYB-OE* lines that underwent GA_4 + 7_ treatment during vegetative growth to reproductive growth stage had the same flowering time as *Col-0*; meanwhile, the GA-treated *Col-0* showed an early flowering phenomenon (**Figure 4A**). This not only indicates that *PtGAMYB* does respond to GA regulation but also suggests that *PtGAMYB* is involved in the regulation of flowering as a regulator of the GA pathway and that sufficient GA accumulation is necessary for normal flowering in plants.

### 3.5 Overexpression *PtGAMYB* leads to pollen developmental defects in *Arabidopsis*

**Figure 5:**
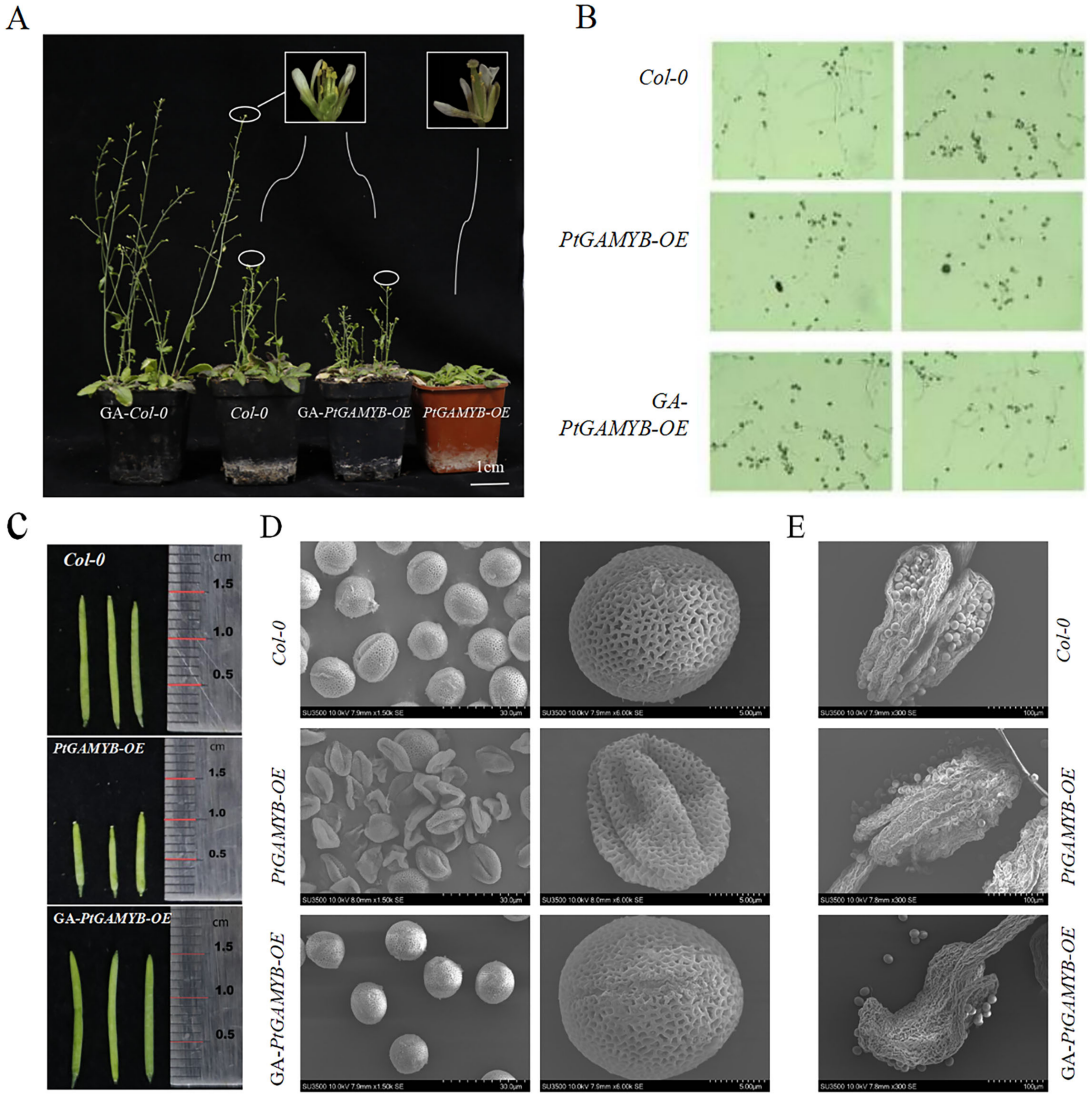
Transgenic phenotypic observations, statistics, and microstructural analysis of *PtGAMYB* in *Arabidopsis*. **(A)** Transgenic phenotypic of *PtGAMYB* in *Arabidopsis*. **(B)** Pollen vitality of *Col-0*, *PtGAMYB-OE*, and GA-*PtGAMYB-OE* lines. **(C)** Pod length of *Arabidopsis*. **(D)** Pollen morphology of different transgenic lines under scanning electron microscopy. Bars=30 µm. **(E)** Anther morphology of *Col-0*, *PtGAMYB-OE*, and GA-*PtGAMYB-OE* lines under scanning electron microscopy. Bars=100 µm.

Ectopic expression of *PtGAMYB* in *Arabidopsis* induced abnormal flower phenotypes (**Figure 5**). Compared with *Col-0* plants, which were able to disperse pollen normally, *PtGAMYB-OE* plants were unable to disperse pollen at stage 13 of floral development (**Figure 5A**). Pollen viability assays revealed severe sporophytic defects in *PtGAMYB*-OE lines. While *Col-0* pollen exhibited 80% germination efficiency with robust tube elongation within 8 h, transgenic pollen displayed sporadic germination (4%) and stunted tube growth (**Figure 5B**). There was a significant difference in the length of fruit pods between *Col-0* and *PtGAMYB-OE* as plants grew and matured (**Figure 5C**). We also found that *PtGAMYB-OE* had defective fruit pods and some seeds did not form. The presence of these phenomena in the fruit pods suggests that *PtGAMYB* overexpression may lead to reproductive abnormalities. Observation of pollen structure showed that *Col-0* plants’ pollen was intact, whereas *PtGAMYB-OE* lines’ pollen showed collapsed pollen walls, deformed pollen structure, and abnormal development (**Figure 5D**). In addition, further observation of the anthers at the 13th developmental stage by scanning electron microscopy revealed that the anthers of *Col-0* were normal, whereas some of the *PtGAMYB-OE* anthers were crumpled, and even for those anthers that were able to dehiscence normally, most of the pollen did not show normal morphology (**Figure 5E**).

Then, pistil development in exogenous GA_4 + 7_-treated *PtGAMYB-OE* plants were observed. Exogenous GA_4 + 7_ treatment during vegetative growth restored mature pollen germination rates in *PtGAMYB*-OE lines to wild-type levels (**Figure 5B**). Scanning electron microscopy results showed that the pollen structure of the GA-treated *PtGAMYB-OE* strain was restored to normal and the aberrant structure was restored, indicating that increasing GA accumulation during the nutrient growth stage could restore pollen stunting and sterility in the *PtGAMYB-OE* strain (**Figure 5D**). Similarly, the GA-treated *PtGAMYB-OE* plants showed restoration of normal anther structure, full pollen grains, and normal pollen wall development (**Figure 5E**). These results suggest that GA regulates pollen formation through *PtGAMYB*, and the signaling pathway of GA-*PtGAMYB* regulating pollen development is more conserved and shares a similar regulatory mechanism with that of the angiosperm pollen formation signaling pathway.

### 3.6 *PtGAMYB* binds directly to the *PtLEAFY* promoter and regulates its expression

**Figure 6:**
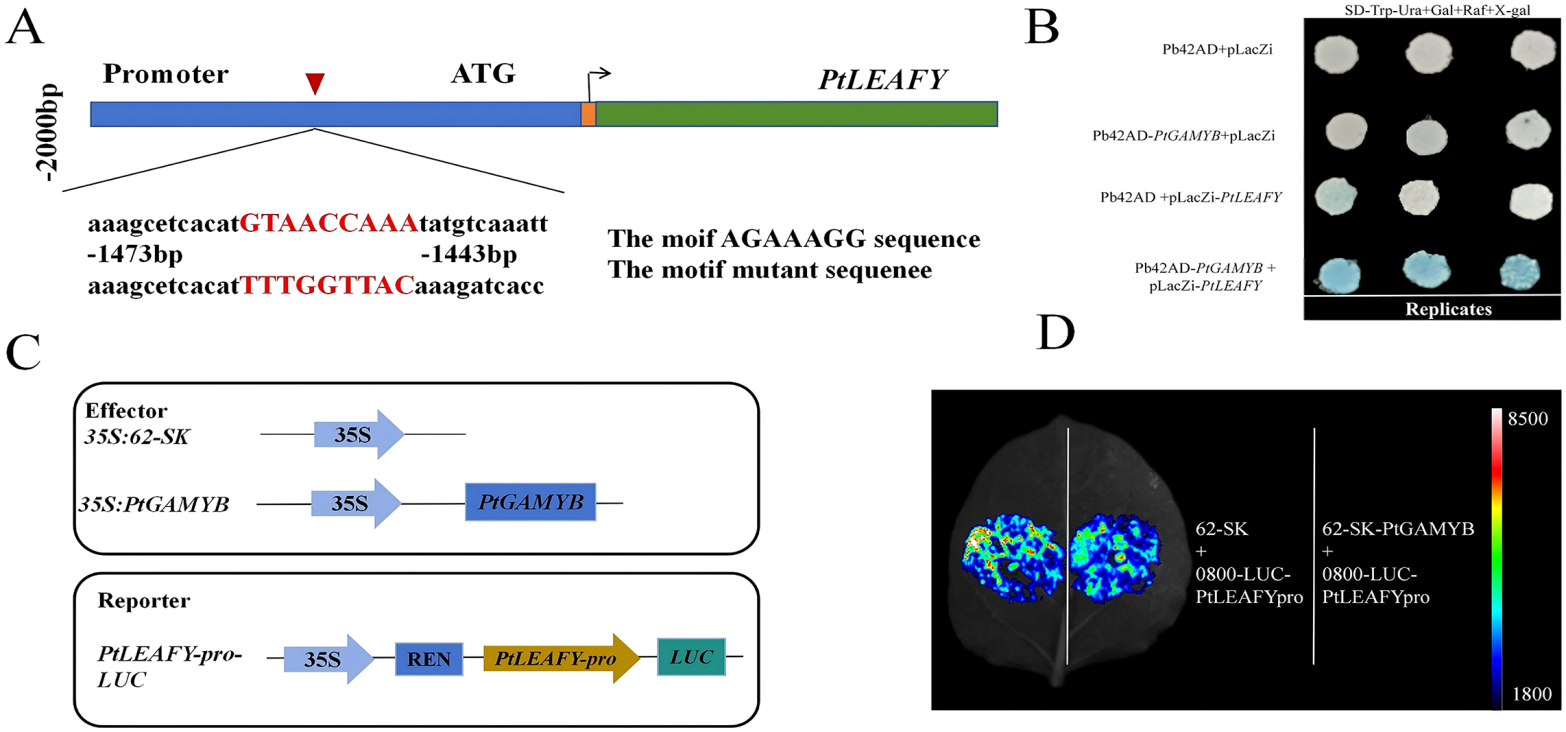
*PtGAMYB* binds directly to the *PtLEAFY* promoter and regulates its expression. **(A)** Motif GTAACCAAA sequence and mutant sequence of *PtLEAFY* promoters. **(B)** Yeast one-hybrid of *PtGAMYB* with the motif GTAACCAAA sequence of *PtLEAFY* promoters. **(C)** Schematic of the effector and reporter structures were used for a luciferase reporter assay. **(D)** LUC assays between the CDS of *PtGAMYB* with *PtLEAFY* promoters.

Previous studies have shown that *PtGAMYB* was expressed at a high level during male cone development, but its expression pattern was opposite to that of *LEAFY* in *Pinus tabuliformis*, which was different from the known proportional relationship between *GAMYB* and *LEAFY* expression in angiosperms (Song et al., 2025). Therefore, we hypothesized that *PtGAMYB* might be a negative regulator of *PtLEAFY*. We first verified whether *PtGAMYB* could regulate the promoter of *PtLEAFY* by yeast one-hybrid (Y1H) assay. The promoter fragments were fused to the prey vector Placz, and pB42AD-*PtGAMYB* was introduced into the EGY48 yeast strain. The results showed that *PtGAMYB* could directly bind to the promoter of *PtLEAFY* (**Figure 6B**). To further confirm this result, the Luc assay was performed in *N. benthamiana* leaves. When co-infiltrated with *PtGAMYB*, the relative LUC: Ren activity of the *PtLEAFY* promoter fused to LUC was reduced compared with the effector control, suggesting that *PtGAMYB* represses the *PtLEAFY* promoter (**Figure 6D**). Collectively, these results suggest that *PtGAMYB* binds to and represses transcription from the *PtLEAFY* promoter. Furthermore, the lfy-1 null mutant exhibited severe defects in floral organ development, including failure to form normal siliques and sterility (Song et al., 2025). These genetic findings suggest that *PtLEAFY* may act downstream of *PtGAMYB*.

### 3.7 PtDPL1 interacts with *PtGAMYB* to inhibit *PtLEAFY* expression

**Figure 7:**
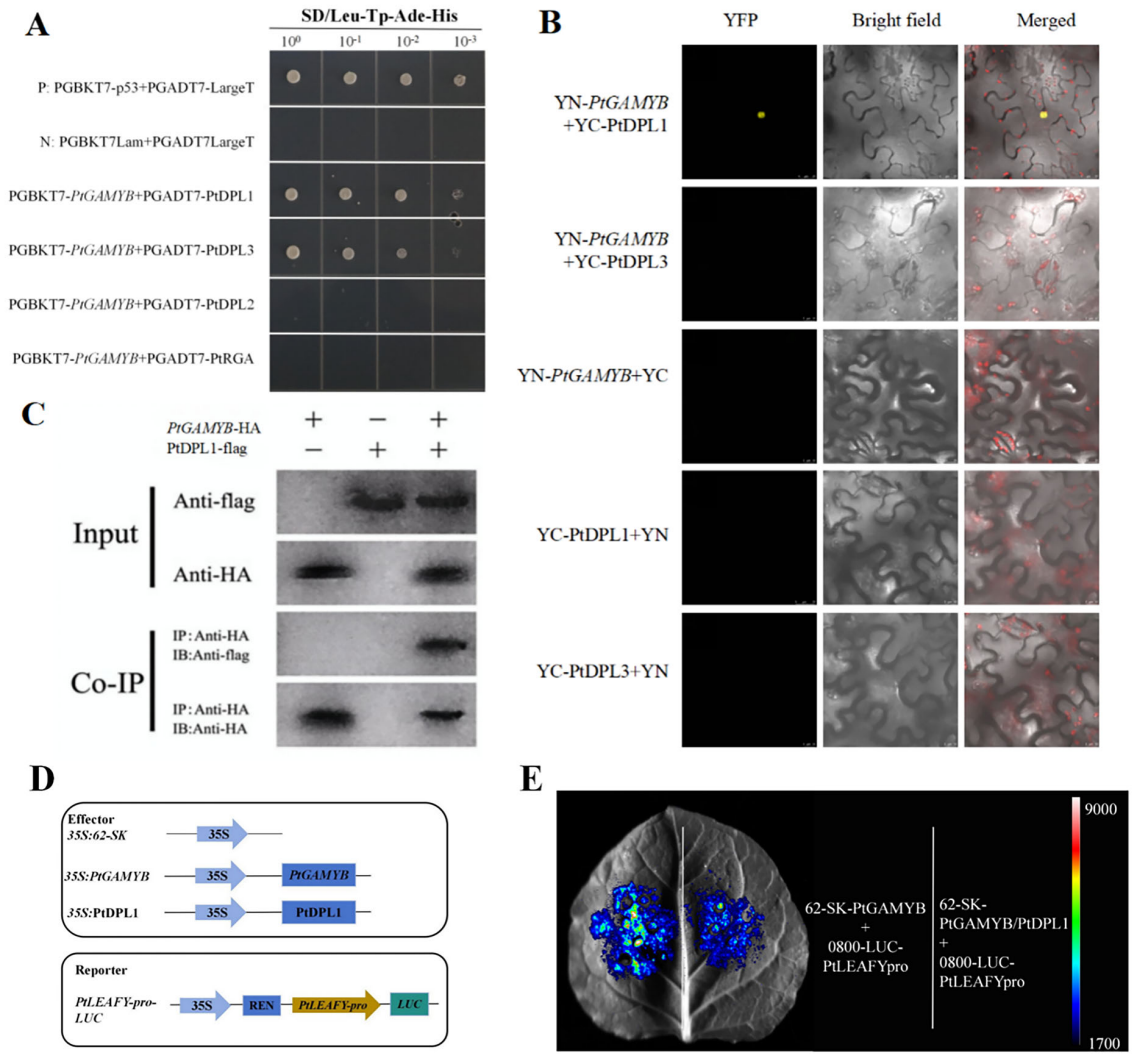
PtDPL1 interacts with *PtGAMYB* to inhibit *PtLEAFY* expression. **(A)** Yeast two-hybrid analysis of *PtGAMYB* binding to PtDELLA in yeast cells. **(B)** BiFC analyses. The fluorescence detected in the nucleus of transformed *N. benthamiana* cells co-expressing *PtGAMYB*-nYFP with PtDPL1-cYFP under the control of the cauliflower mosaic virus (CaMV) 35S promoter (Pro35S). Bars=25  µm. **(C)** CoIP assays. Total proteins were extracted from 8-day-old transgenic Arabidopsis seedlings simultaneously overexpressing PtDPL1 and *PtGAMYB* (*PtGAMYB*-HA+PtDPL1-Flag) under the control of Pro35S. Flag-fused PtDPL1 was immunoprecipitated using an anti-Flag antibody (1:250), and the co-immunoprecipitated *PtGAMYB*-HA protein was detected using an anti-HA antibody (1:10,000). **(D)** Schematic of the effector and reporter structures were used for a luciferase reporter assay. **(E)** LUC assays between the CDS of *PtGAMYB* and PtDPL1 with *PtLEAFY* promoters.

Yeast two-hybrid and coimmunoprecipitation assays were performed to validate if there is a relationship between *PtGAMYB* and PtDELLA. It was found that PtDPL1, PtDPL3, and *PtGAMYB* co-transformed yeast strains grew well on solid SD culture with -Leu-Trp-His-Ade in all dilutions, but PtDPL2, PtRGA, and *PtGAMYB* co-transformed yeast strains did not grow on solid SD culture with -Leu-Trp-His-Ade, supporting that there was a strong mutual interaction between PtDPL1, PtDPL3, and *PtGAMYB* in the yeast cells (**Figure 7A**). To verify the interaction between *PtGAMYB* and PtDPL1 in plant cells, we performed BiFC assays. When PtDPL1-cYFP was co-expressed with PtGAMYB-nYFP in *N. benthamiana*, strong YFP fluorescence was observed in the nucleus, whereas PtGAMYB-nYFP and PtDPL3-cYFP showed no fluorescence (**Figure 7B**). These results reflect the interaction between *PtGAMYB* and PtDPL1. A stable interaction relationship between *PtGAMYB* and PtDPL1 was further identified by co-immunoprecipitation techniques (**Figure 7C**). These results clearly demonstrate that PtDPL1, but not PtDPL2, PtDPL3, and PtRGA, interacts with *PtGAMYB* through direct protein–protein interaction. A dual-luciferase assay further revealed that co-expression of PtDPL1 and *PtGAMYB* significantly suppressed *PtLEAFY* activity (**Figure 7E**). Collectively, these results suggest that PtDPL1 can inhibit *PtLEAFY* expression through direct interaction with *PtGAMYB*.

## 4 Discussion

GA is a key phytohormone involved in stamen development of plants (Jin et al., 2022). *GAMYB*, originally isolated from the barley aleurone layer, is a key component of the GA signaling pathway and plays a critical role in the GA floral regulatory network (Gubler et al., 1999; Washio, 2003; Li et al., 2016). Many angiosperms possess multiple *GAMYB* members, for example, in *Arabidopsis*, wheat, and tomato, and these are highly expressed in anthers, with predominant expression in pollens (Gocal et al., 2001; Wang et al., 2012; Plackett et al., 2011). Numerous studies have shown that *GAMYB* contributes to flower induction, another development, and disease resistance (Plackett et al., 2011). However, further studies are needed to elucidate the characteristics, expression patterns, and functions of *PtGAMYB* in *Pinus tabuliformis*. With the recent release of a high-quality chromosome-level genome of *Pinus tabuliformis* (Niu et al., 2022), a comprehensive analysis of the *PtGAMYB* gene in this species is now possible.

*PtGAMYB* belongs to the *GAMYB* family in *Pinus tabuliformis*, as it possesses the conserved structural features of *GAMYB* proteins and contains three characteristics motifs of the *GAMYB* family. Phylogenetic analysis demonstrated that *PtGAMYB* shares the highest sequence homology with *PkMYB33* from *Larix kaempferi*, both representing conifer *GAMYB* members and implying a potential common ancestral origin. Notably, *PtGAMYB* clustered within the same clade as *AtMYB33* and *AtMYB65* (**Figure 1**). The evolutionary conservation of *PtGAMYB*, particularly its putative upstream role in the GA signaling cascade, merits further exploration.

In this study, 26 GA-associated differentially expressed genes were identified, with a greater number detected during female cone development than in male cones. Among these, *PtGAMYB* consistently exhibited a specific pattern of strong upregulation. Specifically, *PtGAMYB* was predominantly expressed during male cone development in *Pinus tabuliformis*, whereas its expression remained low in female cones. At the early developmental stage, *PtGAMYB* expression levels were similar in male and female cones, but levels increased rapidly in male cones as development progressed. Expression declined steeply before rebounding at the 5th developmental stage, suggesting that *PtGAMYB* is required to maintain high expression throughout male cone differentiation and development, whereas low expression is maintained during female cone development (**Figure 2**). Subcellular localization studies showed nuclear-specific accumulation of *PtGAMYB*-GFP fusion proteins. Fluorescence *in situ* hybridization further revealed tissue-specific expression patterns, with a predominant *PtGAMYB* expression detected in microspores and pollen grains. Quantitative analysis confirmed significantly higher expression in pollen compared with other tissues, strongly supporting a primary role for *PtGAMYB* in pollen development (**Figure 3**). Collectively, these findings collectively provide experimental evidence for the involvement of *PtGAMYB* in regulating microsporogenesis and pollen dispersal mechanisms in pine species.

Previous studies have reported that *GAMYB* regulates flowering time and pollen fertility in plants such as *Arabidopsis*, *Phyllostachys pubescens*, and rice (Gocal et al., 2001; Fleet and Sun, 2005; Li et al., 2013a). In *Arabidopsis*, the *GAMYB*-related genes, *MYB97, MYB101*, and *MYB120*, are highly expressed in mature pollen tube and pollen grain, where they jointly regulate pollen development (Liang et al., 2013). In *Phyllostachys pubescens*, a large amount of malformed pollen was observed during the late stages of pollen development, including surface collapse of deformed pollen grains, reduced activity, and significant decreased pollen germination and seed setting (Hou et al., 2018). In rice, *OsMYB33* acts as a major regulator of tapetum development, pollen wall formation, and cell wall organization (Tsuji et al., 2006). To further investigate the function of *PtGAMYB*, we generated *A. thaliana* overexpression lines (*PtGAMYB-OE*). These lines exhibited delayed flowering, pollen wall collapse, malformed pollen structures, abnormal development, and decreased seed set compared with *Col-0*. These phenotypes are consistent with previous findings in angiosperms such as *Arabidopsis* and rice (Millar and Gubler, 2005; Liu et al, 2010). We then tested whether GA_4 + 7_ treatment could restore the phenotypes of *PtGAMYB-OE* lines. Exogenous GA application successfully rescued flowering time, pollen structure, and pollen activity, restoring normal growth. These results confirmed that *PtGAMYB* is regulated by GA and functions as a regulator of the GA-mediated flowering pathway. Moreover, sufficient GA accumulation is essential for normal flowering in plants (**Figures 4**, **5**). Collectively, our findings suggest that GA regulates male cone formation through *PtGAMYB* and that the GA-*PtGAMYB* regulatory module in conifers is relatively conserved, sharing similarities with the angiosperm pathway controlling pollen development.

To refine the GA regulatory network with *PtGAMYB* as the core, we investigated the interaction between *GAMYB* and DELLA, a key regulator in the GA pathway. Previous studies have shown that the GA-DELLA-*GAMYB* signaling pathway affects the development of stamen and flowering time in many species (Achard et al., 2004; Aya et al., 2009). *PtGAMYB* can be activated by GA and may function as a GA signal transduction factor similar to *AtMYB33*. Under short-day conditions, exogenous application of GA_4_ increases the expression of *GAMYB* and *LEAFY. AtMYB33* is closely associated with the GA-binding region of the *LEAFY* promoter, thereby promoting flower development. This regulatory pattern has been validated in multiple species (Gocal et al., 2001; Aya et al., 2011). In addition, miRNA159 has been found to regulate flowering time indirectly by modulating *LEAFY* activity (Xie et al., 2003; Wang et al., 2018). We observed a strong correlation between the expression patterns of *PtGAMYB* and *PtLEAFY* in the reproductive tissues of *Pinus Tabuliformis*, highlighting the need to determine how *PtGAMYB* responds to GA signaling. In this study, we found that *PtGAMYB* repressed *PtLEAFY* expression and that PtDPL1 can directly interact with *PtGAMYB*, thereby enhancing its repressive effect on *PtGAMYB* on *PtLEAFY* (**Figures 6**, **7**). This regulatory mechanism has not been found in angiosperms and appears to be specific to conifers such as *Pinus Tabuliformis.* We therefore hypothesize that the *GAMYB*-DELLA interaction network in conifers has undergone evolutionary divergence.

**Figure 8:**
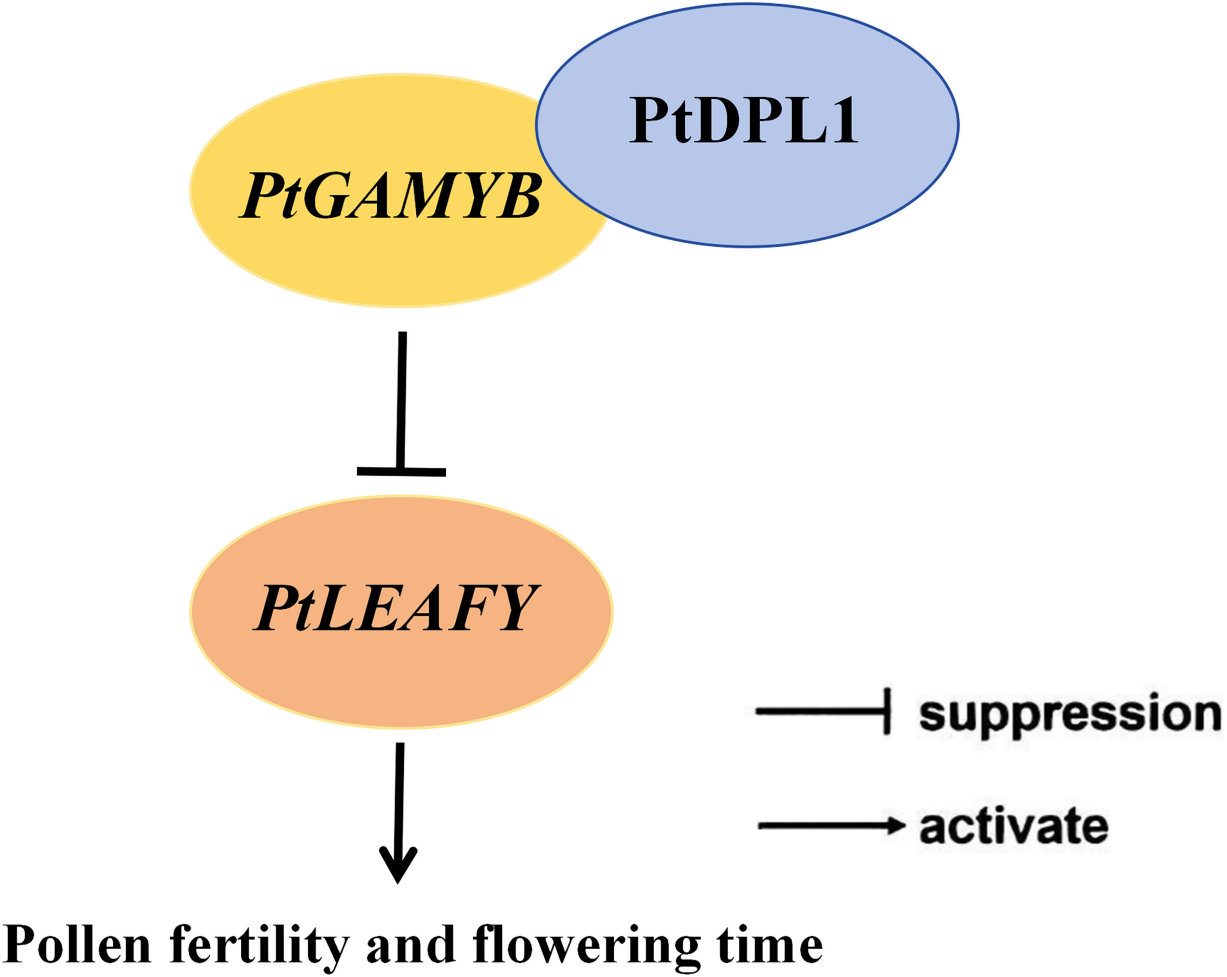
Proposed model of *PtGAMYB* and *PtLEAFY* in regulating flowering in *Pinus tabuliformis*. *PtGAMYB* interacts with PtDPL1 to regulate pollen fertility and delay flowering via suppressing the expression of *PtLEAFY*.

Our study delineates a novel GA signaling module (PtDPL1/*PtGAMYB*-*PtLEAFY*) that is central to reproductive development in *Pinus tabuliformis* (**Figure 8**). By identifying *PtGAMYB* as a regulatory hub within the conifer flowering network, we advanced the mechanistic understanding of GA-mediated reproductive programming in gymnosperms. These findings provide an important reference for elucidating the framework of reproductive development networks in other conifer species.

## Data Availability

The sequenced raw reads generated during the current study have been submitted to the National Center for Biotechnology Information (NCBI) with accession number: SRA056887 (https://www.ncbi.nlm.nih.gov/sra/?term=SRA056887).
