# Supplementary material for: The PtDPL1/PtGAMYB-PtLEAFY module regulates pollen fertility and flowering time in Pinus tabuliformis
Source: Front Plant Sci. 2026 Mar 16;16:1711944. doi: 10.3389/fpls.2025.1711944 (PMC13033750; doi:10.3389/fpls.2025.1711944)
Supplement: Supplementary file 1 [file DataSheet1.docx]

Supplementary Material

**Supplementary Data**

**Supplementary Appendix S1.** CDS sequences, amino acid sequences and promoter sequences of *PtGAMYB.*

*>PtGAMYB*

ATGTGTAATGGTGATGTACTACAAACACCCACAGGGACAATGAGTTCAGAAACGAGTGAAAGTGGGGCAACCAAGGAACAACAAAATCTTGAGGGAGGAGGGGAGGTCATTGGAGGAGGGAATGGGGGTGGTGGGTCTCTGAAGAAGGGGCCATGGACTTCTGCAGAGGACGCAATCCTGGTAGAATATGTGAAGAAGCATGGTGAGGGCAATTGGAATGCAGTGCAGAAACATTCTGGGTTGTTTCGCTGTGGGAAGAGTTGTCGTCTGAGATGGGCTAATCACCTCAGGCCTAATCTCAAGAAGGGTGCCTTCACTGCTGAGGAAGAGCAGATCATCATTGAACTCCATGCCAAGCTCGGAAACAAATGGGCTCGCATGGCTGCCCAGCTGCCTGGGCGGACTGATAACGAGATTAAGAATTACTGGAACACACGGATCAAGAGAAGGCAGAGACAGGGATTGCCTCTTTACCCGCCCGATCTGCCTCTACAGCAATCCAACAGCGAAAATCAACAAAATCAGCAGCCCATCAACGGTGCAGATGCTTCAACGTCCCAGCATAATCATAGTCATAATCATCATGAGTTCCTGTCCGGCTCTGCAAAACTTGAGATTCCTAATGTCACCTTTGATAGTCTGAAACCTAGCCAGCATGCATTGACATACACTTCGTTTCCGGCTTTGACAGAGGTTTCAATGAGTTCGATTCTCAATCAGGCACTGGGATTGTCACAGAGTTACCGTATGGTAAACCCAATTCAGCGTGCAAAGCGGGTTCGGGATAGCGAGAGCATGATGCCCTTTGGTGTAGGAGGAGGAGGGATCTCCCCTTTTGGTCAGTTTGTCGATGACACCCCATTAAAGAGCATAGGGGAACCGTGCTTCAAGACTGCTCGGCGAATGCCATATGCTCAGCCTCCCAGATTGGATGGGTCGTCATATTCTCAGCCTCCCAGATTGGATGGGTTTGGGGCAAATGGTTTTCCTTATGATCCTAATCTGAGCAACAGGAATTCCAATCATAATCTATCAACGCCCCTTGGGGGTTTGATGATTGGCAGCCATGCCCTTTTGAATGGCAATCTCTCTCCTCCTACACCCTTACCAGGTGTGAAGTTGGAGCTCCCTTCAAGCCAACTTGCGGAGTCTGTGCATACCACAGGCACGCGTCTGGAGACAGTGACTACTCCTACAGAAAATAACTCATACACACTGCCCCCTCCTGTTCCATCCAACCATGTTGATGGCTTCTCCCCACGCAACAGTGGGTTGTTAGAAGCTTTGCTGCAGGAGTCTCAAGCAATGGGTGGCGGCGGGAATAAGCGATCCTCGGAAATGATCACACAATTGCCACCAATCTCATCAAGAAGTAACTTGACCAATTGTGTTGGAGCAAGTCAGTCGGAAACAGAATGCGGTGAATACAGTGATCCAATCACCCCTCTGGGTGGTCCTGCCGCTTCAGTATTCAGTGAAAACACACCCCCCTTGAGCACCAGTCCATGGGACGAGTCATCATCCGCTCAGTCAGCAATTGGGGTGAACATAAAGACTGAGGAGCGCAACGAATTTATGTCTAGTACTAACTGTGGAGATGAAGAATTTTCTACGTTATTGAATCTTACAAGGCCAGATGTTTCGCCTGTCTCAGATTGGTATGATTCCCATGCTGAGGTTGTTGAGAAAGAGCAATCTGCTGTTAGTGATGCTCTTGCCACGCTATTCAATGATGATTTCTGTGTAGATATTCAACAGCTGGCTTCAGGACCTTCTACATCAAATCAAGTTTGGGGCCTTGGTTCTTGCCACTGGAATAATATGCCTGGTGTCACACAAATTGTTGATCTTCCTTGA

*>PtGAMYB*

MCNGDVLQTPTGTMSSETSESGATKEQQNLEGGGEVIGGGNGGGGSLKKGPWTSAEDAILVEYVKKHGEGNWNAVQKHSGLFRCGKSCRLRWANHLRPNLKKGAFTAEEEQIIIELHAKLGNKWARMAAQLPGRTDNEIKNYWNTRIKRRQRQGLPLYPPDLPLQQSNSENQQNQQPINGADASTSQHNHSHNHHEFLSGSAKLEIPNVTFDSLKPSQHALTYTSFPALTEVSMSSILNQALGLSQSYRMVNPIQRAKRVRDSESMMPFGVGGGGISPFGQFVDDTPLKSIGEPCFKTARRMPYAQPPRLDGSSYSQPPRLDGFGANGFPYDPNLSNRNSNHNLSTPLGGLMIGSHALLNGNLSPPTPLPGVKLELPSSQLAESVHTTGTRLETVTTPTENNSYTLPPPVPSNHVDGFSPRNSGLLEALLQESQAMGGGGNKRSSEMITQLPPISSRSNLTNCVGASQSETECGEYSDPITPLGGPAASVFSENTPPLSTSPWDESSSAQSAIGVNIKTEERNEFMSSTNCGDEEFSTLLNLTRPDVSPVSDWYDSHAEVVEKEQSAVSDALATLFNDDFCVDIQQLASGPSTSNQVWGLGSCHWNNMPGVTQIVDLP

>*PtGAMYB* Up_Stream_Len 2000

TCAGAATTTGAACCAGTGACTTATCGTAGGGAGTAACTGTGCTTTAGTAAATTGCATGAGCTTGCGAATCTACATGATTTTCTACATGGTATTAGAGCTGAGAACAATATACTAGAGGAGATTTCGAATTTTTGAGATTTGGATTACAAGTGAAATTCGAATTTGCAACACATACTTCAGGGATGACAGAGGGCGTTCTACGGTTTGCGGCTCGAGTATGCCATGGAAGGCAACTTGAATTTTATAGTTCGGAGGGACAAAATGGAGCATGTGCTAGAGGATAATTTCTTGAAGGAATTCGTTGATCAAGAAATTCCTAAGCCAATAGCGTCGGATGCACAGAATTTGGCTGAATGGAAGAAGTGTCTCGAGTCTCCATGGCAAGGGAACTCCGTATGCTATGTGGAAAGCATTGATGGACTTGTATCAGAACAACAGTGACCAAAGGAAATTGGCACTGAAGTACAAACTTTGAAAAATCAAAATGGAGAAAGTCGAGACTATTCCAAAATACTTGACCAAGTTCACTCAATGTCATGATGGACTTCGAAGTGTTGTAATCACAATTGCCAATGATGATATGGTGAGTCTTGCTCTTATAGGACTCCCAAAGAGTTGGCATGGTTATCAGGATTCTATTAAGGGTCGGGAGAAGCTTCCATATTGGGAACGATTGTGGTTGGATTTGATGCAAGAGGAGATCAGGAGGAACACCAGGGATGGCTCTTCATCAAAGACCTTAACAATACATTGATTAACCCATGAGAATTTGAATCAATGAGTTATGGCAGGAAGTTCCTTAATGGTGCACCCGAGGATGGGGCGGCAAAAAATGGGGTTTGTGGTAATGGAGTATTTATTGTATTGAAACTTGGTGATTTTTATCTATTCAGTTGGAACAATGGCGAGGGATTGAATAATAGAGCTGAGCTGTTAGCTCTTTGGGGCATCCTATTTTGTGAAACGTGGCTTTGAATTGTTTCCTTGGACATTTTCAGAGATTCCAAAGTGATTGTAAATTGAGTCTTGGAGAGAACAAATTTTGGCTGGCCAATGCTCATCAACTGAATGCATTGCATGAAATGTTAGAAAAATACTTCCAGAATCTATCCTTTTTACATATCTACATTGAACAGAATCATATAGCAGATGTTTTGTCCAAAGAAAGATGCAACACGTGGCCAGGAAAAATATTATTCTCCACTTCTTACAAAAGGAAATGGCCATGGTTATGGGAACATAAATGTAAATTAGGTAGGTTGTGTTTCTGAACAGCATTTTTGTCCAGTAAGTGTATACTAAAGATATGTAAGTCTGTTTTGGGTGAGGGGTTGAAAGTAGAATGATAATTTTATGTGTGAGATAAGAGATCCATGTACTAGAAAATAGTATAGCCAGCAAAAGGAGGCCCAGTTTTTCTATAAAAAGGCGAGATTCGAAAAATGAGGAAGATATTGATTGGCAAGAATAAGTAAATAACTTTTTTTATAATAGAAAGTTTGTTGGTTTTTTTTTTATTGAAAAAACTACAACAAAATAGTGGGGAAAATTTCTTATTATGAGGTGATTTTATGTTAAATACAGTCAATTTTTTTTCCGTACCTATCAACATTCTTTATCAAATTGATAAAAAATTGATCAAAACATACACTACTAAGACACTAATAAAGTTAGTGACACTGACAAAGTTACTGAAACTGACCAGTGTTTCAGATTTGAAATTCCTACCCTAACAGCACCTATTCAAAATCTCAATCCTGATGTTTTGTCTTGTCCAATTGAGTTGTCAAAACTTTTTCCCAACTCAATAGAGCCATAAAACCTGTACCCCTGATGTTTGAACAAATTTCATTTTCCACTTAAAATCTCTGGAGGTCAATAAATCTATGAATTTCGCCCAAATTCCATTCCAGATTGAATGCCGGCGTTTATCGTGTCGATGAATGAAAGCTCTGCTATTGCTACCCGTCCTCAACGCACTCAGTGACCGGGAAAGGTGA

**Supplementary Table S1.**  List of all primers in this paper.

| **No** | **Gene ID** | **Primer Sequence** |
| --- | --- | --- |
| 1 | *PtGAMYB* | F:ATGTGTAATGGTGATGTACTACAAACACCCACAGGGACAAT |
|  |  | R:AGGAAGATCAACAATTTGTGTGACACCAGGCATATTATTCCAGT |
| 2 | PtGAMYB-GFP | F: CACGGGGGACTCTAGAATGTGTAATGGTGATGTACTACAAA |
|  |  | R:CCCTTGCTCACCATGGTACCAGGAAGATCAACAATTTGTGTGACA |
| 3 | PtGAMYB-BD | F:GAATTCCCGGGGATCCACATGTGTAATGGTGATGTACTACAAA |
|  |  | R:GCAGGTCGACGGATCCTCAAGGAAGATCAACAATTTGTGTG |
| 4 | PtGAMYB-Co | F: TACCGAGCCCGAATTCGTTTTCAAGGAAGATCAACAATTTGTGTG |
|  |  | R:CACCACTTTGTACAAGAAAGGAAGATCAGCGATTTGT |
| 5 | pDONR221 | F:GTAAAACGACGGCCAG |
|  |  | R: CAGGAAACAGCTATGAC |
| 6 | pEarley | F:CAGGAAACAGCTATGAC |
|  |  | R: AGGCACCACTTTGTACAAG |
| 7 | Halo-Seq | F: CTGAATCTGCTGCAAGAAGAC |
|  |  | R: TGCTAGTTATTGCTCAGCGGTG |
| 8 | PtGAMYB-Halo | F:ACTTTCAGAGCGATAACGCGATGTGTAATGGTGATGTACTACAAA |
|  |  | R:TACCGAGCCCGAATTCGTTTTCAAGGAAGATCAACAATTTGTGTG |
| 9 | PtDPL1-AD | F:CATCGATACGGGATCCATGAAGCGACAACACTTTCAATTGC |
|  |  | R:CGAGCTCGATGGATCCTTAACAGCGTTGCCAGGC |
| 10 | PtDPL2-AD | F:CATCGATACGGGATCCATGAAGCGACAACACTTTCAATTGC |
|  |  | R:CGAGCTCGATGGATCCTCAAGAGAGGTTCGTGCCTTTCC |
| 11 | PtDPL3-AD | F:CATCGATACGGGATCCATGGAAAGGGCTGCCAAGG |
|  |  | R:CGAGCTCGATGGATCCCTAGGAGCCTTGCCATGC |
| 12 | PtRGA-AD | F:CATCGATACGGGATCCATGGAAAGGGCTGCCAAGG |
|  |  | R:CGAGCTCGATGGATCCCTAGGAGCCTTGCCATGC |
| 13 | PtDPL1-Co | F:ACAAGTTTGTACAAAAAAATGAAGCGACAACACTTTC |
|  |  | R:CACCACTTTGTACAAGAAACAGCGTTGCCAGGCAGA |
| 14 | PtGAMYB-PYN | F:CGCCACTAGTGGATCATGTGTAATGGTGATGTACTACAAA |
|  |  | R:TACTATCGATGGATCAGGAAGATCAGCGATTTGTGTGA |
| 15 | PtDPL1-PYC | F:CGCCACTAGTGGATCCATGAAGCGACAACACTTTCAATTGC |
|  |  | R:TACTATCGATGGATCCACAGCGTTGCCAGGCAGA |
| 16 | PtDPL3-PYC | F:CGCCACTAGTGGATCCATGGAAAGGGCTGCCAAGG |
|  |  | R:TACTATCGATGGATCCGGAGCCTTGCCATGCAGATG |
| 17 | PtGAMYB-62SK | F:CGGGCTGCAGGAATTCATGTGTAATGGTGATGTACTACAAA |
|  |  | R:GCTTGATATCGAATTCAGGAAGATCAGCGATTTGTGTGA |
| 18 | PtDPL1-62SK | F:CGGGCTGCAGGAATTCATGAAGCGACAACACTTTCAATTGC |
|  |  | R:GCTTGATATCGAATTCACAGCGTTGCCAGGCAGA |
| 19 | PtLEAFY-0800 | F:CGGGGGATCCACTAGTTGTTTTTGTTTCACATTAAAAGGAC |
|  |  | R:CCGCTCTAGAACTAGTCAATATCTGCTACTCAAATAGTG |
| 20 | PtGAMYB-Pb42AD | F:TGCCTCTCCCGAATTCATGTGTAATGGTGATGTACTACAAA |
|  |  | R:CGAGTCGGCCGAATTCTCAAGGAAGATCAGCGATTTGTG |
| 21 | PtLEAFY-pLacZ2µ | F:ATCTGTCGACCTCGAGTGTTTTTGTTTCACATTAAAAGGAC |
|  |  | R:GAGCACATGCCTCGAGCAATATCTGCTACTCAAATAGTG |
| 22 | actin12 | F:GGCTGACACCATCACCAGAAC |
|  |  | R:GTTGGTCGCCCTCGTCATACT |
| 23 | *PtGAMYB-qPCR* | F:TCGCTGTGGGAAGAGTTGTC |
|  |  | R:GGGCATCATGCTCTCGCTAT |

**Supplementary Table S2.** Identification of *PtGAMYB* in *Pinus tabuliformis.*

| Gene name | Gene ID | Amino acid length （aa） | Protein Mol. Wt. （kDa） | pI |
| --- | --- | --- | --- | --- |
| *PtGAMYB* | Pt5G01300.1 | 1857 | 66.65KDa | 5.5 |

**Supplementary Table S3.** Differential gene expression of GA synthesis and signal transduction pathway

| **target_id** | **F1** | **F2** | **F3** | **F4** | **F5** | **F6** | **M1** | **M2** | **M3** | **M4** | **M5** | **M6** |
| --- | --- | --- | --- | --- | --- | --- | --- | --- | --- | --- | --- | --- |
| *PtGA3ox1* | 0.000 | 0.000 | 0.416 | 1.569 | 3.954 | 0.311 | 0.000 | 0.000 | 0.000 | 0.322 | 0.000 | 0.000 |
| *PtGA3ox2* | 0.730 | 0.110 | 0.324 | 2.496 | 1.929 | 1.271 | 0.636 | 1.352 | 0.872 | 1.640 | 0.921 | 0.000 |
| *PtGA2ox1* | 0.696 | 3.375 | 4.661 | 8.210 | 8.218 | 7.755 | 4.502 | 0.299 | 0.892 | 2.766 | 2.268 | 1.672 |
| *PtGA2ox2* | 27.527 | 27.645 | 29.653 | 22.579 | 13.833 | 9.027 | 34.809 | 12.186 | 21.299 | 14.396 | 4.657 | 3.149 |
| *PtGA2ox3* | 30.442 | 52.884 | 43.999 | 43.404 | 44.597 | 45.633 | 40.313 | 36.345 | 36.524 | 38.767 | 38.956 | 36.835 |
| *PtGA2ox4* | 1.232 | 0.000 | 0.000 | 0.000 | 0.000 | 0.000 | 0.000 | 0.000 | 0.000 | 0.000 | 0.000 | 0.000 |
| *PtGA2ox5* | 0.601 | 0.443 | 0.812 | 0.637 | 1.222 | 0.549 | 0.246 | 0.305 | 0.103 | 0.118 | 0.127 | 0.139 |
| *PtGA2ox6* | 7.551 | 20.037 | 21.590 | 27.287 | 25.763 | 11.246 | 11.399 | 3.731 | 13.426 | 4.805 | 2.245 | 0.092 |
| *PtGA2ox7* | 0.005 | 6.752 | 0.010 | 3.116 | 9.955 | 1.872 | 0.000 | 0.046 | 0.000 | 0.024 | 0.000 | 0.001 |
| *PtGA2ox8* | 33.785 | 23.484 | 22.078 | 25.914 | 26.577 | 49.706 | 27.551 | 14.159 | 21.520 | 8.151 | 2.490 | 1.331 |
| *PtGA2ox9* | 2.085 | 2.452 | 10.196 | 5.740 | 6.368 | 14.666 | 0.000 | 1.895 | 1.258 | 3.380 | 2.012 | 0.182 |
| *PtGA2ox10* | 85.970 | 97.137 | 122.090 | 152.545 | 166.885 | 189.091 | 137.142 | 137.968 | 174.263 | 125.426 | 25.375 | 28.715 |
| *PtGA2ox11* | 22.778 | 36.909 | 34.964 | 27.419 | 27.736 | 27.167 | 9.424 | 13.287 | 11.762 | 17.163 | 14.536 | 9.575 |
| *PtGA2ox12* | 4.873 | 2.785 | 1.491 | 4.074 | 7.088 | 16.525 | 5.375 | 25.592 | 24.919 | 24.177 | 48.089 | 65.704 |
| *PtGA20ox1* | 2.573 | 0.087 | 0.000 | 1.430 | 2.358 | 1.683 | 1.910 | 0.000 | 0.000 | 0.811 | 0.172 | 0.000 |
| *PtKAO1* | 92.703 | 55.301 | 65.232 | 49.403 | 26.215 | 41.952 | 126.940 | 17.693 | 22.330 | 20.586 | 3.579 | 0.373 |
| *PtKAO2* | 1.093 | 4.906 | 3.013 | 3.561 | 7.430 | 3.746 | 5.206 | 1.321 | 2.074 | 2.414 | 0.852 | 0.673 |
| *PtKO1* | 0.000 | 0.428 | 0.425 | 1.845 | 4.738 | 0.670 | 0.349 | 0.000 | 1.813 | 5.142 | 2.003 | 0.167 |
| *PtKS1* | 0.547 | 0.139 | 2.997 | 1.101 | 0.000 | 2.940 | 1.360 | 1.126 | 2.943 | 0.018 | 0.000 | 1.065 |
| *PtCPS1* | 0.683 | 10.429 | 13.578 | 27.043 | 20.497 | 2.779 | 1.332 | 8.860 | 12.873 | 8.231 | 0.929 | 0.495 |
| *PtGID1* | 35.976 | 56.824 | 60.702 | 39.238 | 42.154 | 50.582 | 21.557 | 36.355 | 31.068 | 54.675 | 57.210 | 68.784 |
| PtDPL | 8.037 | 7.596 | 10.104 | 15.872 | 11.031 | 15.444 | 7.473 | 6.865 | 2.715 | 3.088 | 2.258 | 2.222 |
| PtRGA | 40.991 | 30.434 | 39.103 | 33.507 | 30.429 | 31.903 | 25.652 | 18.760 | 16.312 | 21.292 | 11.442 | 13.947 |
| *PtGAMYB* | 0.584 | 3.121 | 6.416 | 2.382 | 7.663 | 8.156 | 1.178 | 24.437 | 36.837 | 45.806 | 48.367 | 45.536 |

**Supplementary Table S4.** The expression level of *PtGAMYB* under different tissues.

| **Gene name** | **Gene ID** | **needle** | **root** | **vegetative bud** | **male_cone** | **Female_cone** | **pollen** |
| --- | --- | --- | --- | --- | --- | --- | --- |
| *PtGAMYB* | Pt5G01300 | 2.81 | 9.52 | 5.08 | 50.15 | 16.93 | 87.78 |


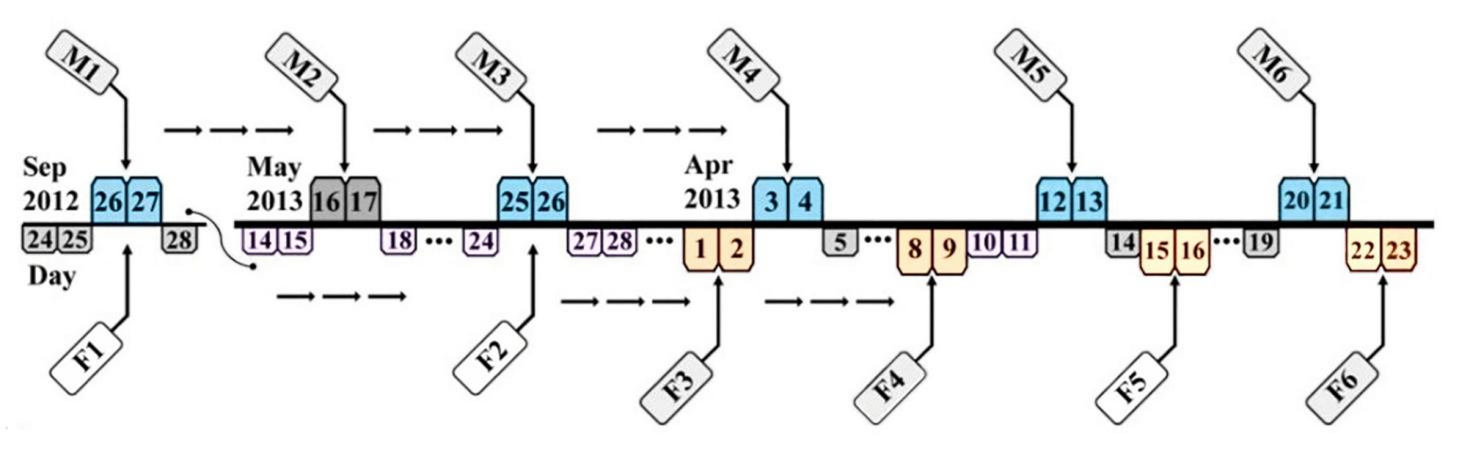


**Supplementary Fig S1.** Timeline of sampling at different stages of reproductive development in male and female cones, the graphing is referenced to Niu et al (2016).


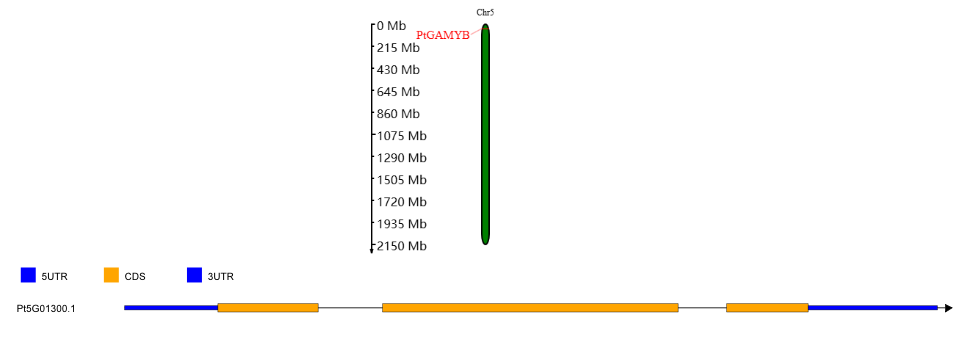


**Supplementary Fig S2.** Chromosome localization and gene structure of *PtGAMYB.*


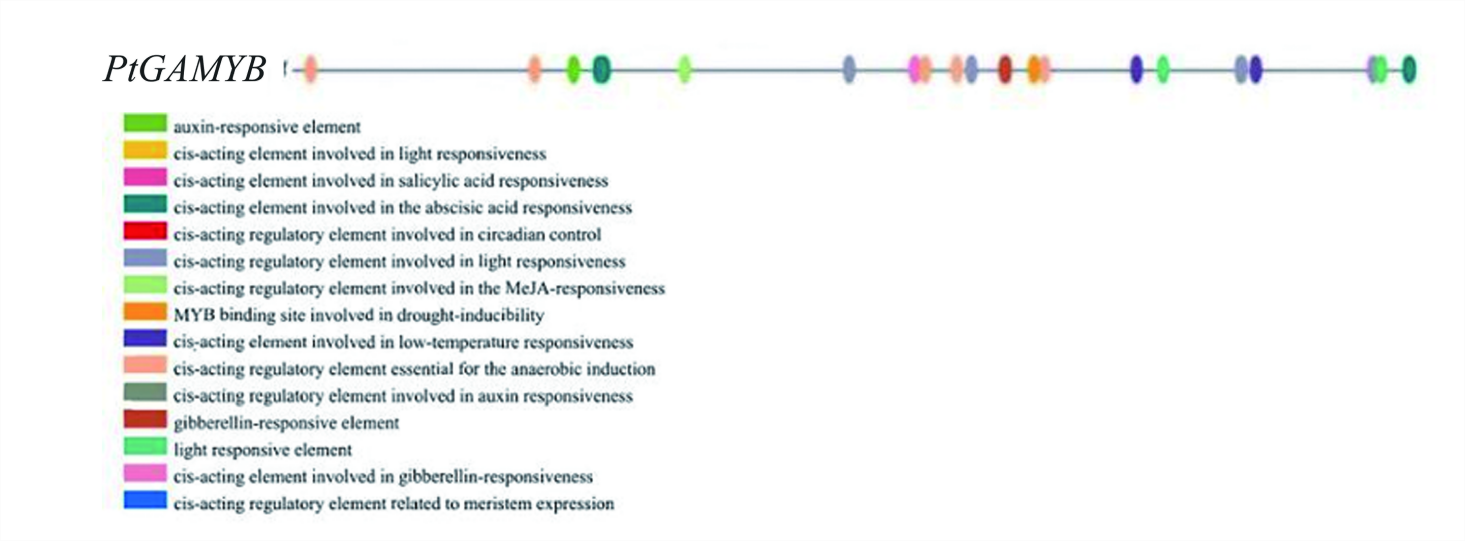


**Supplementary Fig S3.** Promoter cis-acting elements of *PtGAMYB.*

*
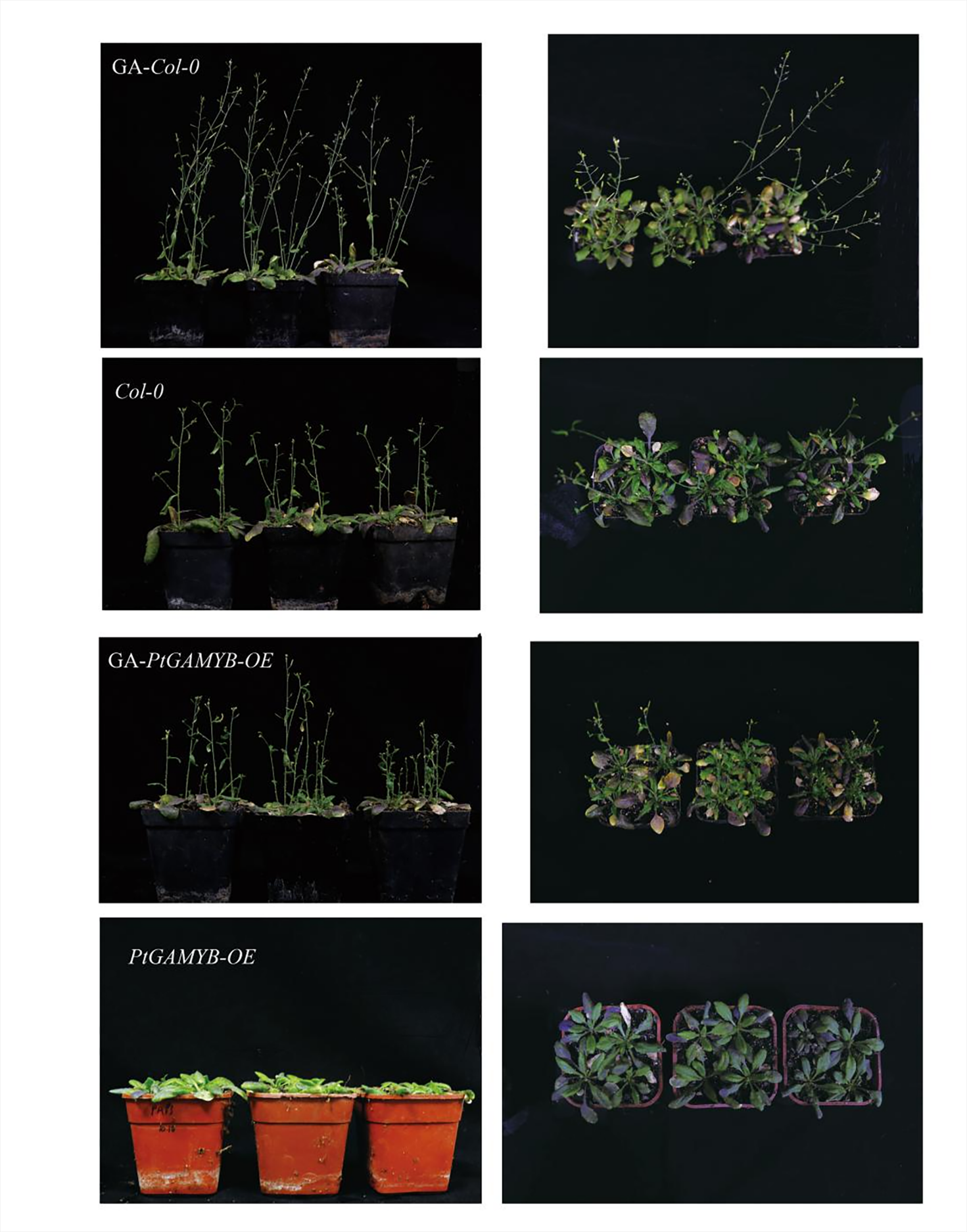
*

**Supplementary Fig S4.** The phenotype of *PtGAMYB-*OE in *Arabidopsis*.
